# The effects of apoptosis inhibitor of macrophage in kidney diseases

**DOI:** 10.1186/s40001-023-01597-3

**Published:** 2024-01-04

**Authors:** Yixia Cao, Boyan Hu, Yunhe Fan, Wei Wang, Mingxuan Chi, Moussa Ide Nasser, Kuai Ma, Chi Liu

**Affiliations:** 1 School of Medicine, University of Electronic Science and Technology of China, Chengdu, China; 2 Department of Nephrology, Sichuan Academy of Medical Science and Sichuan Provincial People’s Hospital, Sichuan Renal Disease Clinical Research Center, University of Electronic Science and Technology of China, Chengdu, China; 3 Chinese Academy of Sciences Sichuan Translational Medicine Research Hospital, Chengdu, China; 4 Reproductive & Women-Children Hospital, School of Medical and Life Sciences, Chengdu University of Traditional Chinese Medicine, Chengdu, China; 5 Department of Cardiac Surgery, Guangdong Provincial People’s Hospital (Guangdong Academy of Medical Sciences), Guangdong Cardiovascular Institute, Southern Medical University, Guangzhou, 510100 Guangdong China; 6 Department of Nephrology, Osaka University Graduate School of Medicine, Osaka, Japan; 7 Renal Department and Nephrology Institute, School of Medicine, Sichuan Provincial People’s Hospital, University of Electronic Science and Technology of China, Sichuan Clinical Research Center for Kidney Diseases, Chengdu, China

**Keywords:** Apoptosis inhibitor of macrophages, Kidney diseases, Chronic kidney disease

## Abstract

Kidney disease is a progressive and irreversible condition in which immunity is a contributing factor that endangers human health. It is widely acknowledged that macrophages play a significant role in developing and causing numerous kidney diseases. The increasing focus on the mechanism by which macrophages express apoptosis inhibitor of macrophages (AIM) in renal diseases has been observed. AIM is an apoptosis inhibitor that stops different things that cause apoptosis from working. This keeps AIM-bound cell types alive. Notably, the maintenance of immune cell viability regulates immunity. As our investigation progressed, we concluded that AIM has two sides when it comes to renal diseases. AIM can modulate renal phagocytosis, expedite the elimination of renal tubular cell fragments, and mitigate tissue injury. AIM can additionally exacerbate the development of renal fibrosis and kidney disease by prolonging inflammation. IgA nephropathy (IgAN) may also worsen faster if more protein is in the urine. This is because IgA and immunoglobulin M are found together and expressed. In the review, we provide a comprehensive overview of prior research and concentrate on the impacts of AIM on diverse subcategories of nephropathies. We discovered that AIM is closely associated with renal diseases by playing a positive or negative role in the onset, progression, or cure of kidney disease. AIM is thus a potentially effective therapeutic target for kidney diseases.

## Introduction

### Kidney disease

Kidney disease is a significant and increasing global challenge for public health. Scientists found that the incidence of chronic kidney disease (CKD) in China reached 10–13% in 2015 [1]. A study published in 2017 indicated that kidney diseases almost contributed to 3.0% of deaths in South Asia, and CKD would become more rapid [2]. In addition, epidemiological characteristics of kidney diseases are heterogeneous and are affected by age, population, region, economic development, and other factors. Moreover, patients who are suffering from diabetes, hypertension, high uric acid, and obesity have a higher risk of developing kidney diseases with a worse prognosis. Regulating the abnormal immune response in vivo and preventing the kidney from being attacked by autoantibodies is one of the most effective methods to treat kidney diseases. However, it may produce adverse effects on normal tissues simultaneously. More extensive and deeper research from new perspectives is needed to further our cognition of kidney diseases and provide more significant treatments for them.

### Apoptosis inhibitor of macrophage (AIM)

AIM, also known as CD5-like antigen (CD5L) and Api6, which has a molecular weight of 40 kDa, is principally produced by tissue macrophages, including liver Kupffer cells. It is well-known that AIM is a homolog of human Spα and belongs to the scavenger-rich receptor cysteine (SRCR) superfamily (30), which consists of proteins expressed by both adaptive and innate immune cells. Besides, AIM contains an SRCR domain composed of approximately 100 amino acids and traits as highly conserved and rich cysteine [3, 4]. The gene AIM is highly conserved in mammals, representing similar functions [5]. However, it can also exhibit subtle differences in different host cell types, which register as unique structures and functions [6].

It was initially recognized that AIM is an apoptosis inhibitor that supports macrophage survival against apoptosis-inducing stimuli. Later, the inhibitory effects of AIM on apoptosis in myeloid, lymphoid, and other cells were successively discovered [6]. Unfortunately, the molecular mechanism that AIM regulates apoptosis has not been elucidated. However, there have been some findings that may provide useful insights into how AIM regulates apoptosis. On one hand, AIM can relieve the inhibition of anti-apoptotic factors. The research illustrated that AIM could alleviate the inhibitory effect on the anti-apoptotic function of insulin-like growth factor-I (IGF-I) via binding to insulin-like growth factor binding protein-4 (IGFBP-4) [7]. As a result, AIM plays an important role in immunoregulation by holding back the apoptosis of immunity cells. On the other hand, AIM can mitigate the occurrence of inflammatory reactions. It has been reported that AIM may indirectly regulate inflammation or phagocytosis by binding the targets with toll-like receptors (TLRs) or scavenger receptors in pattern recognition receptors (PRRs) [4]. Meanwhile, with the synergistic effect of CD36, AIM can enhance autophagy and retard inflammatory cascades induced by active TLRs by reducing TNF and IL1B while increasing IL10 expression [8]. In addition, AIM can also induce anti-obesity effects in fat tissues by combining with fatty acid synthase (FASN) and reducing its activity, promoting lipolysis [9].

There is an interesting phenomenon in the blood that the ratio of aim to five-line IgM is close to 1:1. The latest research shows that the IgM pentamer with a large interspace about an angle of 50 degrees obviously differs from the structure of the symmetrical pentagon model [10]. Therefore, the gap inside the pentamer is large, providing a specific room for fixing AIM [11–13]. The pentamer consists of five IgM monomers and an additional polypeptide, which functions as a joining chain (J chain) [11–13]. Besides, the monomer IgM is composed of four polypeptide chains, including two heavy chains and two light chains. These chains are constituted of 14 immunoglobulin domains, which contain two groups of VH–Cμ1–Cμ2–Cμ3–Cμ4/VL–CL [12]. The J chain attached two monomers on both sides of the IgM space at the cysteine Cys575 sites on the IgM Fc domain tail, and the rest of the adjacent monomers also bind together at the same position [12, 14]. The five monomers attach each other tightly via the extra disulfide produced by the Cys414 sites on the IgM monomer Fc domain (contained Cμ2–Cμ3–Cμ4 chains and the short tail region), constituting an asymmetric pentagon that owns a space at an angle of 50 degrees [12, 15].

The AIM possesses three SRCR domains, namely, SRCR1, 2, and 3, which can attach with other targets [16, 17]. The SRCR 2 and SRCR 3 separately promote disulfide bonds and the surface charge effects to connect with the Fc domain on IgM. Afterward, the lateral IgM–Fc and AIM form the Fc domains in the gap edges through these effects [12]. In addition, IgM pentamers cannot traverse the kidney glomerular filtration barrier and enter the urine, because their size is approximately 1000 kDa. Therefore, the filtration of AIM can be avoided by combining with IgM to expand the molecular size itself, thus increasing the plasma half-life time of AIM. Under stable conditions, AIM mainly remains at relatively high concentrations (approximately 5 μg/mL) in the human body [13, 17].

In the blood, AIM is stored in the form of IgM–AIM. In contrast, IgM-free AIM (called free AIM) will release active AIM when it is needed [13, 17]. Studies have demonstrated that the size of AIM will reduce by almost 10 kDa after free AIM is rapidly ruptured in the blood [17]. Besides, after the Lys264 site is replaced by alanine, AIM cannot be excreted by the kidney. It demonstrates that the Lys264 site could be the cleaved site in the free AIM SRCR3 domain [17]. In addition, studies found that these ruptured free AIMs (called small AIM, sAIM) will be preferentially excreted into the urine and no longer bind to IgM, thus preventing the continuous increase of AIM level in the blood, especially the level of functional AIM [17]. As it was demonstrated, compared with the original free AIM, the debris removal function of sAIM is enhanced. Therefore, it is very unlikely that excessive accumulation of AIM in the blood will lead to harmful results when AIM is used to treat disease [17]. It is worth noting that the correlation between AIM and IgM might affect the binding of IgM with the Fcα/μR at follicular dendritic cells (FDCs) surface to induce less internalization of IgM, thereby promoting the retention of IgM [13]. More autoantigens and autoantibody binding are produced when IgM is deposited on FDCs, making it more possible to accelerate the progression of autoimmunity [13]. The above evidence reminds us that AIM–IgM, the inactive form of AIM, contributes greatly to the occurrence and development of immune diseases. Therefore, we should pay more attention to it; more research is needed to advance this intriguing field.

### AIM and various disease

AIM is considered a double-edged sword for the human immune system, which has different effects on the progress and prognosis of the disease. For example, the CD5L/AIM protein expressed by retinal pigment epithelium cells can induce the clearance of oxidized low-density lipoprotein (oxLDL). However, its function may be destroyed by anti-CD5L/AIM auto-antibodies, resulting in age-related macular degeneration [18].

In addition, AIM circulation level may be a biomarker, potentially diagnosing some diseases. Recent studies illustrated that the increased prostate-specific antigen (PSA) ratio to AIM is highly correlated with prostate cancer recurrence [19]. In addition, the level of circulating AIM can be used to predict mortality in critical patients with Sepsis [20, 21]. Similarly, AIM can be recognized as a diagnostic marker for Crohn's disease, because it can promote intestinal inflammatory response by inhibiting the apoptosis of macrophages [22].

AIM is related to the pathogenesis of many important organ-related diseases. For example, previous studies illustrated that AIM could reduce the occurrence of hepatic steatosis in normal hepatocytes, thereby delaying the onset of hepatocellular carcinoma (HCC) [23]. Besides, the increase of alveolar macrophages (AM) worsens chronic obstructive pulmonary disease (COPD), which was associated with the expression of AIM induced by smoking [24]. In addition, AIM can increase the incidence of inflammation in patients after myocardial infarction [25]. Beyond that, recombinant AIM (rAIM) can be used as a new therapeutic strategy for kidney diseases, such as acute kidney injury (AKI) [26].

Among these organ-related diseases, more and more studies have emphasized that AIM plays an essential role in kidney-related diseases, and the mechanism of AIM in kidney disease is gradually clarified. This article discusses the relationship between AIM and various kidney diseases and tries to find a new way to prevent and treat kidney diseases.

### AIM and AKI

**Fig. 1:**
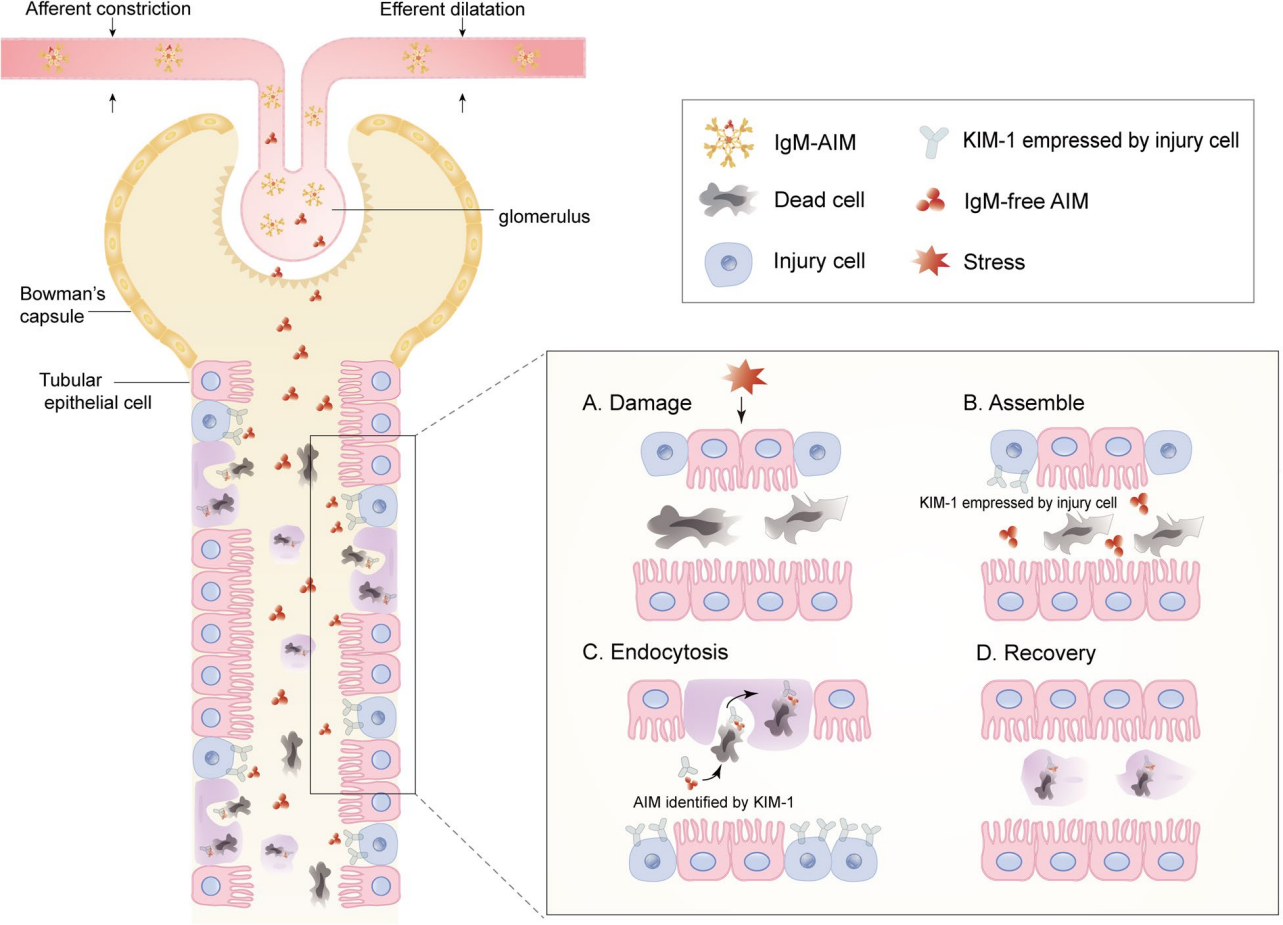
AIM in AKI:** A** proximal renal tubules are damaged by harmful stress, which leads to the deaths of renal PTECs. Afterward, there was a significant amount of desquamated epithelial in the proximal tubular lumen. **B** Injure cells empress KIM-1, which is essential in identifying AIM. In the meantime, circulating AIM concentrations increase significantly. **C** AIM is identified by KIM-1 and combined with luminal fragments. Then, the conjugate is endocytosed by 'semi-professional' phagocytes, originating from the injured but surviving renal PTECs. **D** AIM cooperates with KIM-1 to immediately remove dead cell fragments and promote the removal of fragments in the tube, which is the key to the recovery of AKI

AKI is a frequent clinical complication characterized by the abrupt decline of renal function after exposure to various stressors, including ischemia–reperfusion, nephrotoxins, and sepsis [27]. Of note, AKI is a major public health concern of increased proportions owing to the significantly growing prevalence and high mortality rates [28]. Damage of renal proximal tubule epithelial cells (PTECs) leads to the accumulation of debris in the lumen, which is one of the prominent factors in the progression of AKI [4] (Fig. 1A). Subsequently, debris generated by epithelial cell damage of the proximal renal tubule accumulates in the cavity, leading to increased pressure in the lumen, inducing inflammation and fibrosis, further expanding tissue damage, and decreasing renal function. Therefore, it is important to remove dead cell debris promptly to alleviate inflammatory response in AKI. Recently, several studies reported that AIM is crucially involved in the clearance of intraluminal debris by injured renal tubular epithelial cells (TECs), which is credited with having great potential in alleviating the progress of AKI [29]. Consequently, AIM may provide a novel therapeutic strategy for treating AKI.

According to existing studies, AIM is supposed to play a regulative role in developing acute inflammation [26]. It can be concluded into two aspects: (1) endowing epithelial cells with a phagocytic phenotype and clearing debris in the lumen in time. (2) Regulating the process of lipid metabolism and playing a coordinating role in the process of inflammation alleviation.

In AKI, injured but surviving renal PTECs have been observed to function as ‘semi-professional’ phagocytes, accelerating the clearance of dead cells [30]. In addition, due to the small number of macrophages or dendritic cells in the renal lumen, dedifferentiated epithelial cells are considered the main cell type to clear dead cell debris in the lumen. In this procedure, kidney injury molecule (KIM)-1, also known as TIM-1, encoded by the Hepatitis A virus cellular receptor 1 gene, is markedly upregulated in the proximal tubule. It can induce damaged epithelial cells to differentiate into phagocytes and clear dead cells by recognizing phosphatidylserine and oxidized lipoproteins on the surface of dead cells. In the ischemia–reperfusion injury (IRI) mouse model lacking the KIM-1 mucin domain, the kidney injury was more severe than that of the wild type, accompanied by debris accumulation in the lumen, NF-κ B activity increased, and inflammatory response enhanced [31]. During renal IRI, KIM-1 binds with G protein α 12 directly and inhibits its activation by reactive oxygen species to accelerate the clearance efficiency of apoptotic cells [32]. Similarly, one recent research demonstrated that MUC1 maintains the activity of KIM-1 by competing with KIM-1 for the cleavage of ADAM17 and plays a protective role in the mouse model of IRI [33]. A bioinformatics analysis found that checkpoint kinase 1 and STAT3 are key upstream regulators of KIM-1 expression after AKI [34]. Although all of this evidence indicates that KIM-1 has multiple protective effects in AKI, its exact role has remained inexplicit, mainly because its counterpart has not been discovered [35]. Of note, scientists discovered AIM, also known as CD5L, as a ligand of KIM-1 closely related to the clearance of intraluminal debris by damaged epithelial cells induced by KIM-1 after AKI. Subsequently, AIM dissociates from IgM during AKI, leading to a comparable level of sAIM within promoting to scavenge intra-tube debris [17, 35] (Fig. 1B, C). More studies showed that injection of rAIM could alleviate AKI, significantly improving renal tubular injury and mouse survival. Therefore, rAIM is considered a promising therapeutic option for AKI. In addition to dead cells, AIM can identify a wide range of internal pathogens through its pattern recognition properties, including various toxins. In conclusion, AIM acts as a "recognize-and-destroy" weapon, produced from macrophages and wiping cell debris in systemic circulation [29]. However, due to a lack of research data, the mechanism that AIM facilitates the clearance of debris in the renal cavity after a renal injury has not yet been elucidated.

In adipose tissue, AIM can reduce the cytoplasm's fatty acid synthase (FAS) activity to influence adipocyte status and induce inflammation [36]. AIM can also influence the inflammatory response of monocytes to bacterial surface molecules lipopolysaccharide and lipoteichoic acid by repressing TNF secretion [8]. Functional studies in macrophages demonstrated a positive feedback loop between AIM and some specialized lipid mediator biosynthesis [37]. Furthermore, AIM has been suggested as a marker of plasma-derived vesicles in mass spectrometry [38]. Although there is a lot of evidence that AIM is inextricably associated with the changes in lipid metabolism during acute inflammation, its research in AKI is still insufficient. However, it has been reported that AIM may play a coordinating role in the inflammation alleviation process by changing the lipid mediators' structure in the ALI mice model [26]. Therefore, the relationship between AIM and the changes in lipid metabolism in AKI is worthy of further in-depth study.

In summary, we believe that artificial activation of AIM, such as causing AIM to dissociate from IgM or administering AIM directly, may be employed as a technique for clinical therapy of AKI.

### AIM and CKD

Chronic kidney disease (CKD) is a common disorder with a steadily increasing prevalence over several decades [39]. Since the high incidence, poor prognosis, and high medical cost, CKD has already been an enormous global public health burden [40]. However, there is a lack of effective treatments to alleviate renal tissue injury and renal dysfunction caused by CKD. It is noteworthy that a series of common risk factors, such as hypertension, obesity, and diabetes, can cause CKD. Therefore, it is a great challenge for clinicians and researchers to create effective therapeutic procedures to protect renal function and promote patients' quality of life with CKD. Recently, a series of studies have found that AIM is a potential target for CKD treatment.

Epidemiological studies have illustrated strong and consistent associations between AKI and incident CKD and the progression of CKD [41]. However, there is not enough evidence to prove the causality. Some scholars considered that, under certain conditions, after AKI occurs, the renal repair function is not adapted to inflammation, fibrosis, and sparse blood vessels, resulting in continuous cell and tissue dysfunction and, ultimately CKD [42]. Some studies also confirmed that AIM is important in the process of AKI evolving into CKD. Due to the unique positively charged amino acid cluster, feline AIM–IgM binding affinity is 1000 times higher than in mice [43]. In the process of AKI, the AIM encoded by the cat CD5L gene is not easy to separate from IgM, and the migration of AIM to urine is reduced, failing to clear necrotic cell debris and obstruction of proximal tubules, thus hindering the recovery of AKI and increasing the risk of patients from AKI to CKD [43].

**Fig. 2:**
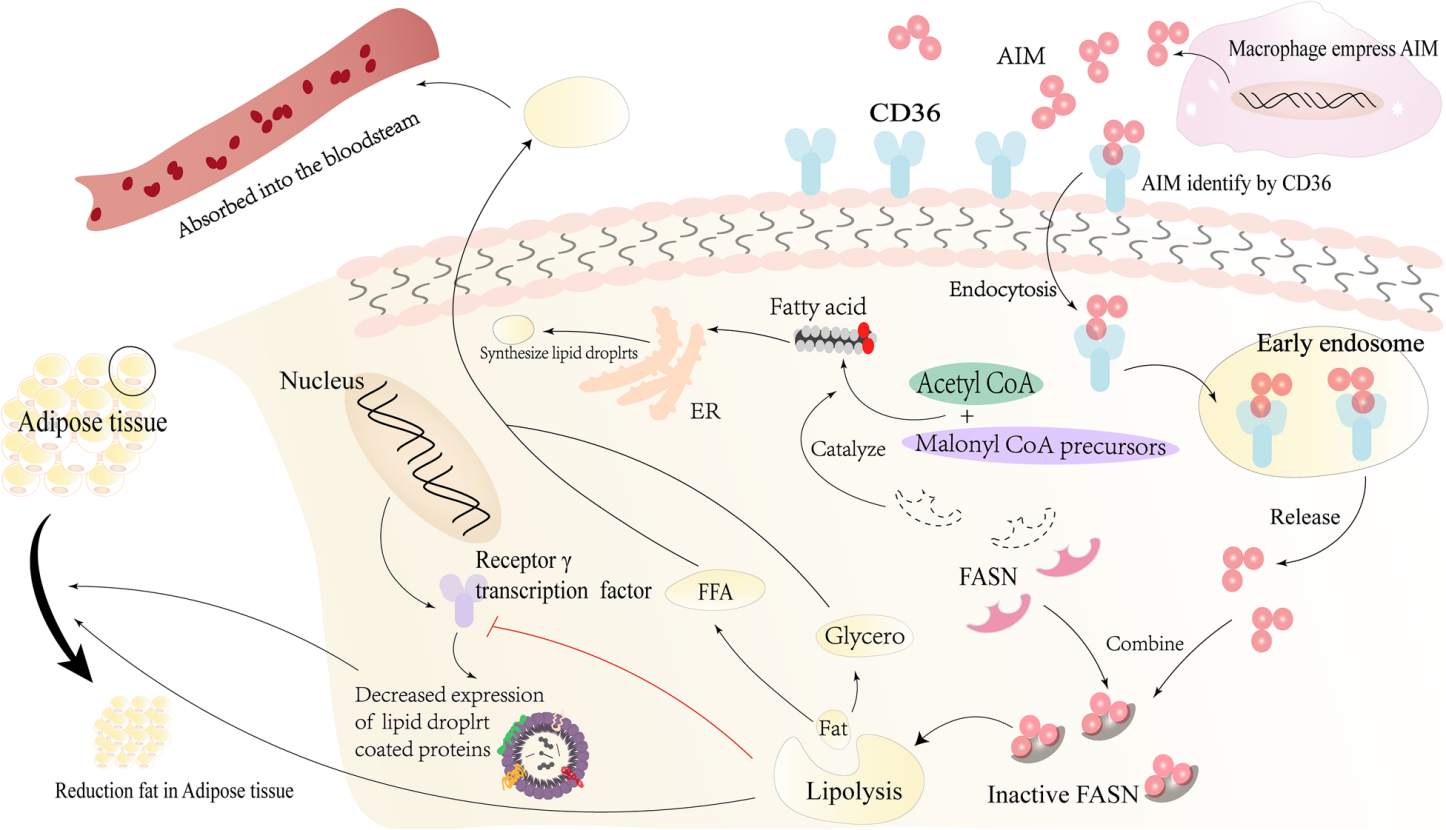
AIM induces lipolysis:AIM is phagocytized into adipocytes via endocytosis induced by CD36. Afterward, AIM combines with FASN to reduce its enzyme activity, which leads to reduced lipolysis and lipid droplet storage. Meanwhile, the lipolysis induced by AIM can reduce the transcriptional activity of receptor γ, which leads to the decrease of protein expression coated by lipid droplets, such as fat adipose-specific protein 27 and perilipin. The above two mechanisms related to AIM work together to reduce the deposition of triacylglycerol in adipocytes and induce the reduction of adipocyte size

However, not all CKD is developed from AKI. Some other hazardous factors, such as obesity and obesity-related diseases, can also lead to CKD [44]. Likewise, research demonstrates that AIM can play an anti-obesity role by regulating lipid metabolism. FASN has the activity of catalyzing the synthesis of palmitate and other saturated fatty acids from acetyl CoA and malonyl COA precursors and is mainly expressed in adipose tissue. FASN binds to IgM-free AIM, which is phagocytized to adipocytes via CD36, to reduce FASN activity, thus inhibiting lipid synthesis and reducing lipid droplet storage [36] (Fig. 2). Meanwhile, receptor γ is a principal transcription factor during adipocyte differentiation and peroxisome proliferator-activated receptor. The transcriptional activity of receptor γ can be reduced by AIM-induced lipolysis, leading to less expression of proteins coated by lipid droplets, such as adipose-specific protein 27 and perilipin [45, 46] (Fig. 2). The above two AIM-related mechanisms of action together reduce the deposition of triacylglycerol in adipocytes and reduce the size of adipocytes [9, 17, 47] (Fig. 2).

Otherwise, AIM can also be considered a potential target for treating other obesity-related diseases and a method that can ameliorate the progression of CKD in a long-term vision. Notably, it has been emphasized that the association of IgM–AIM in obesity contributes a lot to the production of autoantibodies [48]. However, Toshihiro Kai's team artificially synthesized the Fc part of the soluble human immunoglobulin IgM heavy chain. Like natural IgM, it can form a pentamer with J chain and effectively bind AIM in vitro, but its affinity is relatively low, equivalent to antibody–antigen interaction. When injected intravenously into mice lacking circulating IgM, synthetic Fc can protect AIM from renal excretion and maintain the circulating level of AIM [47]. It is gratified to find that because the synthesized FC lacks an antigen recognition variable region, it does not cause unnecessary immune responses. Therefore, we can infer that synthetic Fc could be applied to control obesity invalidly and to treat certain diseases caused by low AIM [47]. In addition, it has been found that AIM deficiency remarkably ameliorated insulin resistance in fat mice. It has been reported that AIM-dependent lipolytic response can induce the outflow of free fatty acids, and the free fatty acids stimulate toll-like receptor 4 (TLR4) [49]. TLR4 activates TNFα receptors to generate inflammatory cytokines, adipokines, and chemokines and are recruited into adipose tissue with inflammatory infiltration of macrophages, increasing insulin resistance [49, 50].

If CKD patients have peritoneal dialysis (PD), AIM also prevents and repairs PD-associated fungal peritonitis models. AIM mediates caspase-1 activation and the production of IL-1 and IL-18, thus contributing to the prevention and treatment of PD-associated fungal peritonitis [51, 52]. 

In addition, the studies revealed that AIM significantly contributes to advancing nephrosclerosis to CKD. Nephrosclerosis is a comprehensive pathological condition characterized by progressive arteriosclerosis in the kidney, followed by ischemic conditions in the glomeruli and tubules, ultimately compromising renal function [53]. Atherosclerosis, a critical precursor to nephrosclerosis, is distinguished by the buildup of cell detritus and lipid deposits in the arterial walls; these changes are caused by two pathophysiological variables of the utmost importance: hyperlipidemia and inflammatory processes [45]. By inhibiting apoptosis and promoting macrophage accumulation in nephrotic tracts, AIM induces inflammation and worsens the progression of atherosclerotic plaque to nephrosclerosis [9, 53].

Besides, research has demonstrated that human AIM in macrophages can influence macrophage adhesion to endothelial ICAM-1 by increasing the expression of macrophage-1 (Mac-1) antigen and integrin LFA-1. ICAM-1 then modifies the appearance of the monocyte that adheres to the arterial endothelial surface and migrates to the subendothelial site, thereby causing an atherogenic injury [45, 54].

Furthermore, research has demonstrated that oxidative stress induces a surge in reactive oxygen species (ROS) within the bloodstream, subsequently elevating ox-LDL levels, which are predominantly detected in the renal tubules. OxLDL binds to LXR/RXR in nuclear receptors following phagocytosis in macrophages; this interaction stimulates the production or secretion of AIM protein in macrophages; in other words, oxLDL induces AIM protein expression [49, 52]. AIM then promotes CD36-mediated oxLDL uptake by binding to CD36, an 88 kDa transmembrane glycoprotein and one of the major scavenger receptors of oxLDL. Hence, we postulate that the interaction between CD36 and AIM could potentially facilitate the uptake of oxLDL, thereby stimulating the lipid uptake of foam cells and contributing to the development of nephrosclerosis [54].

To summarize, AIM can impede macrophage apoptosis, thereby facilitating their adhesion to vascular endothelial cells and fostering the development of foam cells. Nonetheless, foam cells will accelerate the expression of AIM even further, resulting in the eventual formation of a vicious cycle that promotes the progression of nephrosclerosis. It is undeniable that AIM accelerates the process of CKD by facilitating the development of nephrosclerosis. In contrast, glycosylation in AIM can delay the progression of CKD in other ways, which has been confirmed convincingly [55]. SRB1, functioning as a cholesterol transporter, exerts a protective influence on the life cycle of macrophages in atherosclerotic diseases [56]. All of the evidence above suggests that AIM significantly contributes to the progression of CKD, which can be attributed to various factors. It will be highly promising as a treatment to prevent and delay the progression of CKD. Further research is required to resolve the knowledge deficit regarding the biological mechanisms underlying the association between AIM and CKD.

Potential therapeutic applications for nephrosclerosis may include the inhibition of interstitial fibrosis through future rational regulation of cholesterol uptake and excretion factors in conjunction with AIM expression downregulation. The specific etiological mechanism of CKD thus dictates the practical impact of AIM.

### AIM and diabetic kidney disease

Diabetic nephropathy (DN) is the leading cause of end-stage renal disease (ESRD), which occurs in 20–50% of patients with diabetes [56]. At present, with the increasing incidence of diabetes, the incidence of DN is increasing annually [57]. Although the present management of patients with DN, which encompasses blood glucose and blood pressure control, may impede the onset of DN, it cannot avert its progression to end-stage renal failure. Given the economic burden and low quality of life associated with DN, understanding its molecular causes to identify new strategies and additional therapeutic targets is crucial for effective intervention and prevention.

DN often progresses slowly over many years, and it is difficult to predict its process [56]. The current assessment and monitoring are the urinary albumin:creatinine ratio (ACR) and the blood-estimated glomerular filtration rate (eGFR). Although these tests can independently determine a person's renal function, their diagnosis is sometimes contradictory and is of little value in predicting the patient's disease progression [58]. Considering the prognostic limitations of ACR and eGFR, people have been paying attention to alternative biomarkers. AIM is one of the most popular biomarkers that do not rely on eGFP and ACR to predict the rapid decline of renal function in patients with diabetic nephropathy [59].

Macrophages, as the predominant innate immune cells in DN, are commonly observed in the glomeruli and interstitium in experimental DN models and clinical trials at all stages of DN [60]. Accordingly, an experimental result illustrated that the concentration of free AIM in patients with type 2 diabetes mellitus with confirmed kidney disease was 25% higher than in patients without kidney disease [61]. However, some DN patients with rapidly declining renal function have low baseline circulating levels of AIM. Scholars believe some patients with type 2 diabetes may have defects in the expression of the AIM gene [59]. The low level of AIM concentration makes these patients prone to having adverse kidney effects after suffering from diabetes. It is also said that some non-IgM combine with AIM to limit the release of free AIM, which weakens its role in protecting the kidney from sustained renal injury and accelerates the elimination of AIM by the kidney and the metabolism of AIM [62]. This is consistent with the increase of eGFP during the progress of DN [63].

### AIM and glomerular inflammation in IgA nephropathy

IgA nephropathy (IgAN) is one of the most common glomerulonephritis around the world, characterized by the deposition of immunoglobulin A (IgA)-based immunocomplex in the glomerular mesangium [64]. Even though the progress of IgAN is slow, up to 50% of patients develop end-stage renal disease, which is also the main reason for end-stage renal disease in young people [65]. The mechanism of disease development after IgA deposition has not been fully clarified, and no effective disease-specific therapy has been established [64]. A recent study shows that AIM may help to form stable immune complexes in the glomerulus, thus promoting the progress of IgAN [66].

**Fig. 3:**
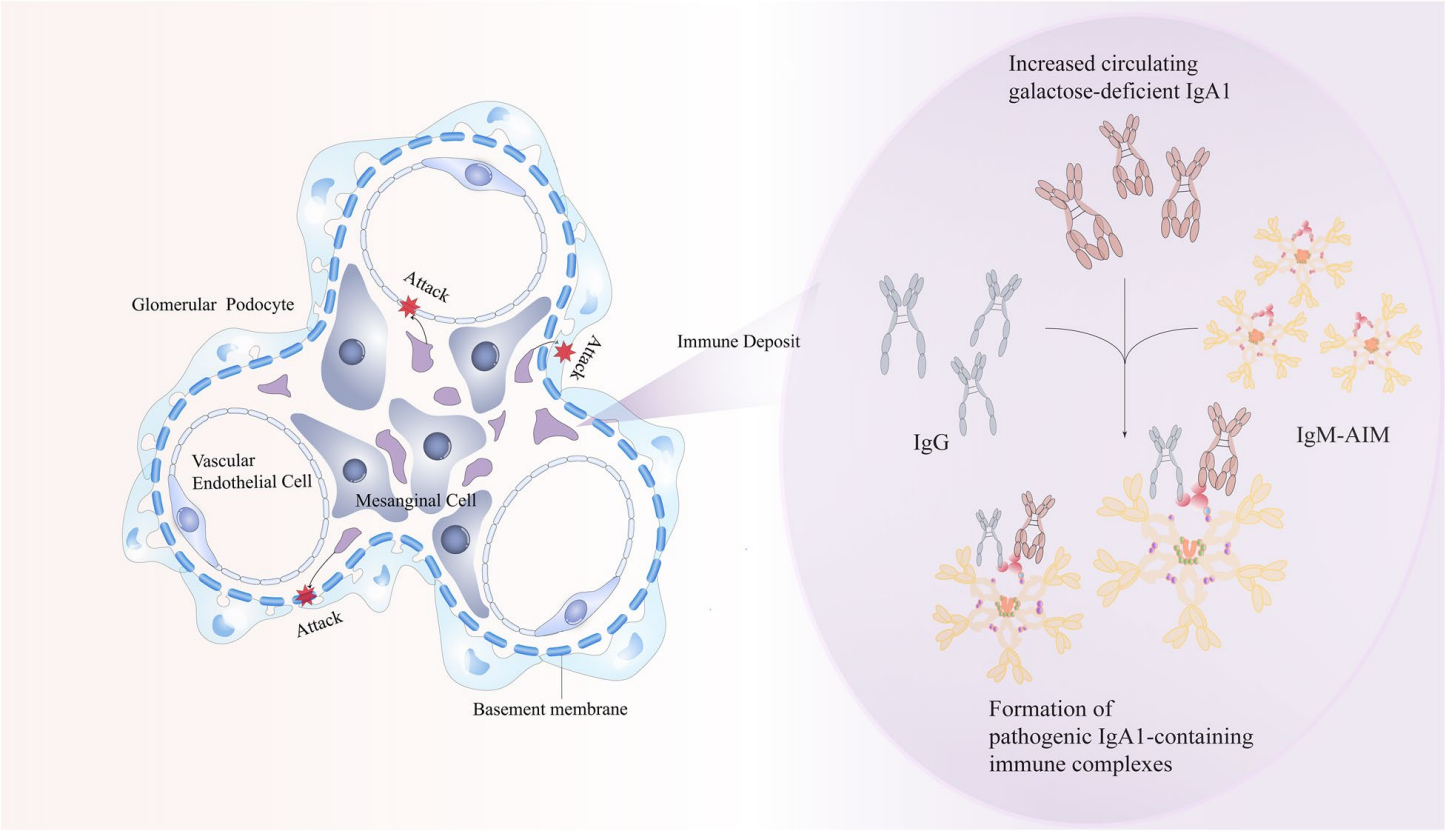
AIM in IgAN:AIM is stably associated with IgM by embedding the missing part of the hexagon in circulating blood. As for the binding mode of AIM to IgA in blood, it mostly binds to IgA–Fc through immunoprecipitation in mice, while the binding mechanism of AIM and IgA in humans is different from that in mice, which is speculated to be related to the abnormal glycosylation and glycosylation formation process of IgA1. We can confirm that IgA and IgM are linked via AIM, but the pathway by which IgG binds to AIM in immunoprecipitation is not clear

The deposition of polymeric IgA1 is one of the most significant histological features widely observed in the kidney of patients with IgAN. It can form an immune complex with variable IgG, IgM, and complement 3 at the glomerular mesangial region [67]. Two stages can be distinguished during the process of IgAN:

- (1) deposition of IgA.
- (2) immune complexes' formation and complements' activation, leading to glomerular damage. Simple IgA deposition cannot induce IgAN. In contrast, the formation of IgA and IgM/IgG immune complexes is the key factor in inducing IgAN, which is closely related to the prognosis and severity of patients [66]. AIM-deficient IgAN mice showed IgA deposition similar to that of wild-type mice but without glomerular accumulation, and subsequent regional inflammation of IgM/IgG/complement, glomerulosclerosis, proteinuria, and hematuria were avoided [66]. This demonstrates that AIM plays an essential role in the formation mechanism of IgA and IgM/IgG immune complexes but has nothing to do with the deposition of IgA. Interestingly, no accumulation of AIM was observed in the kidneys of patients with minimal change in nephrotic syndrome or membranous nephropathy [66]. Therefore, AIM plays an important role in the occurrence and development of IgAN(Fig. 3).

In conclusion, the dissociation of AIM may provide a new therapeutic strategy for IgAN by destroying immune complexes. However, many problems remain to be solved, such as the mechanism through which AIM mediates the combination of IgA and IgM/G. Therefore, more studies are needed to fill the gap in the mechanism and provide more targets for the treatment of IgAN.

### AIM and delayed graft function (DGF) of kidney transplantation

Among the consequences of kidney transplantation, cold and warm IRI injury is inevitable, and long-term ischemia–reperfusion injury may lead to delayed recovery and graft function destruction [68]. Since graft dysfunction is common after kidney transplantation, it will increase the incidence rate and medical burden [69].

ATP depletion and reactive oxygen species production induce apoptosis, particularly necrosis of the renal TECs, during renal IRI. Dead cells lose membrane integrity and release intracellular damage-related molecular patterns (DAMPs) to the extracellular environment, triggering inflammation and promoting cell death [68]. This positive feedback loop of inflammation and cell death or necroinflammation exacerbates tissue damage and potential allo- and non-alloimmune injury [70]. The ability to limit DAMP release from the damaged tissue is essential in the setting of allogeneic transplantation. In a murine model, we found that the enhanced phagocytic clearance of necrotic cell debris within the injured grafts mediates the anti-inflammatory effect of recombinant AIM. Besides, We were pleased to find that grafts from mice treated with recombinant macrophage apoptosis inhibitors (rAIM), which belongs to IgM-free AIM, showed a significant reduction in tubular cell death, tissue damage, as well as local and systemic inflammation [68]. Therefore, AIM plays an essential role in protecting the kidney after transplantation and preventing the rapid decline of its function. In the future, rAIM may become a potential therapeutic drug for reducing DGF after renal transplantation.

## Discussion

In summary, the occurrence and development of kidney diseases are more or less related to AIM, which is a promising research direction. AIM is a human Spα homolog and belongs to the SRCR superfamily. We summarize several common nephrotic subclasses associated with AIM and discover that although the research on AIM in renal diseases is insufficient, its importance to various kidney diseases is undeniable (Table 1). However, there are still many problems to be solved in the research. At present, the research on AIM is still in its infancy. In blood, AIM and pentameric IgM are stably bound by structural matching, while free-AIM is a substance with biological activity. However, in some cases, AIM and IgM have synergistic harmful effects on renal function, and their overexpression in the glomerulus will further aggravate the production of proteinuria. It still perplexes what mechanisms regulate the combination and separation of AIM and IgM and their role in kidney diseases.

**Table 1:** AIM and kidney diseases

Type of kidney disease	Predisposition	Characteristics	Key to cure diseases	The role of AIM	References
AKI	Ischemia– reperfusion	Abrupt impairment of kidney filtration function	Dead cell debris in the lumen	AIM removes cellular debris in the systemic circulation	[4, 17, 27, 29, 35]
CKD	AKI, obesity, nephrosclerosis, diabetes	The blood, the urine, and GFR were abnormal	Dead cell debris, lipid droplets, oxLDL level, and glycosylation	IgM-free AIM cleans the lumen-obstructing debris, decreases triacylglycerol deposition within adipocytes, and facilitates oxLDL uptake	[9, 15, 17, 29, 36, 42, 44, 45, 49, 52–55]
DN	Metabolics, genetic, hemodynamic and environmental factors	High blood pressure, edema, foam uria, proteinuria	Microvascular lesions, morphological and structural changes in the kidney	Free AIM is limited to release, and AIM is elevated in whole plasma	[62, 63]
Glomerular Inflammation in IgAN	Infection, unbalanced immunity, genetics	Glular mesangial cell hyperplasia, and mesangial matrix hyperplasia	IgA with aberrant glycosylations and the deposition of an immune complex which consists of IgA1, IgM, variable IgG, and complement 3 at the glomerular mesangial region	IgA and IgM/IgG immune complexes linked by AIM cause severe inflammatory immune responses	[64, 66, 67]
Delayed graft function of kidney transplantation	Cold and warm IRI	Minuria, anuria, and elevated blood creatinine	Apoptosis and/or necrosis of the renal TECs. Intracellular DAMPs are set free, potentiating allo- and non-alloimmune injury	AIM eliminates necrotic cell debris, limits DAMP release from the damaged tissue, and decreases alloimmunity	[68, 70]

During AKI, AIM separates from IgM into the urine to remove the cell debris and reduce the chance of transition to CKD. In this condition, AIM can also recognize a broad spectrum of internal pathogens besides dead cells, including various toxins, via its pattern recognition property [3]. Besides, free AIM can also be used to reduce tissue damage in kidney transplantation. Beyond that, AIM also possesses an anti-obesity effect that can reduce triacylglycerol deposition in adipocytes. Binding to IgM limits the release of free AIM, thereby impairing its beneficial effect on sustained renal injury. A new strategy to improve circulating AIM can be used as the basis for treating some diseases caused by low AIM. In addition, the classic role of AIM is to inhibit the apoptosis of macrophages, and then the infiltrating macrophages produce various cytokines or chemokines to stimulate the resident immune cells of the kidney, leading to kidney damage. However, more studies have shown that AIM sometimes has a protective effect on the kidney. For example, in AKI, AIM can accelerate the clearance of renal cells in renal tubules, alleviate inflammatory damage, and protect the kidney. Therefore, it is of great significance to clarify in which cases AIM plays a protective role and accelerates the disease's progress. At the same time, what is the action mechanism of AIM in these diseases, and how does AIM interact with other substances that play an important role in kidney diseases? Some studies have shown the benefits of rAIM in treating renal inflammation, which still needs to be convincingly proved in more experimental data and clinical trials.

All in all, there is no doubt that the relationship between AIM and kidney disease is a remarkable research field. However, there are still many unclear contents in this field. Hopefully, this review can stimulate further research in this field and provide new ideas for treating kidney disease.

## Data Availability

Not applicable.

## Abbreviations

ACR: Albumin:creatinine ratio
AIM: Apoptosis inhibitor of macrophage
AKI: Acute kidney injury
AM: Alveolar macrophages
COPD: Chronic obstructive pulmonary disease
CD5L: CD5-like antigen
CKD: Chronic kidney disease
DN: Diabetic nephropathy
DAMPs: Damage associated molecular patterns
eGFR: Estimated Glomerular Filtration Rate
FAS: Fatty acid synthase
FDCs: Follicular dendritic cells
HCC: Hepatocellular carcinoma
IGFBP-4: Insulin-like Growth Factor Binding Protein 4
IGF-I: Insulin-like growth factor-I
IgM: Immunoglobulin M
IgAN: IgA nephropathy
IRI: Ischemic Reperfusion Injury
KIM: Kidney injury molecule
oxLDL: Oxidized low density lipoprotein
PD: Peritoneal dialysis
PRRs: Pattern recognition receptor
PSA: Prostate-specific antigen
PTECs: Proximal tubule epithelial cells
ROS: Reactive oxygen species
sAIM: Small AIM
SRCR: Scavenger receptor cysteine-rich
TECs: Tubular epithelial cells
TLRs: Toll-like receptors
